# Development of Multi-Bioactive Driven Composite Plant Extracts and Functional Study in Mice and Piglets

**DOI:** 10.3390/antiox15040468

**Published:** 2026-04-09

**Authors:** Xin Tao, Yongming Li, Shujie Liu, Wanyun Wu, Jie Wu, Xiaoming Men, Bo Deng, Ziwei Xu

**Affiliations:** Institute of Animal Husbandry and Veterinary Science, Zhejiang Academy of Agricultural Sciences, Hangzhou 310021, China; xindragon@126.com (X.T.);

**Keywords:** composite, plant extract, antibacterial, antioxidant, anti-inflammatory, mice, piglets

## Abstract

This study aimed to develop a multi-bioactive composite plant extract as an alternative to dietary antibiotics for application in animal production. Five plant materials were initially selected from 23 candidate plants via in vitro antibacterial, antioxidant, and anti-inflammatory screening, and formulated into three candidate extracts (C1, C2, C3) by orthogonal design, with respective dominant activities and moderate the other activities. Three feeding trials in mice demonstrated that administration of 1000 mg/kg C1 or C2 caused no adverse effects on hematological parameters or organ indexes. Supplementation with 250 mg/kg C1 or 125 mg/kg C2 significantly increased body weight gain and feed intake, reduced the feed-to-gain ratio, and modulated gut microbiota composition. In LPS-challenged mice, C1 and C2 restored jejunal villus height and crypt depth, downregulated the gene expression of *TLR4*, *TNF-α*, *NF-κB*, and *IL-1β*, and increased hepatic T-AOC activity while decreasing MDA content. Furthermore, a feeding trial in piglets demonstrated that dietary supplementation with 200 mg/kg C2 achieved growth performance comparable to that of conventional antibiotic supplementation, highlighting its potential as a substitute for feed antibiotics. In conclusion, this study has developed a new multi-bioactive composite plant extract that may serve as a promising alternative to feed antibiotics.

## 1. Introduction

The global ban on antibiotics in animal feed has stimulated increasing research interest in alternative strategies. Among these alternatives, phytogenic products are one of the most extensively investigated due to the absence of bacterial resistance development and drug residues. Numerous plant extracts have been reported to exhibit broad-spectrum antibacterial, anti-inflammatory, and antioxidant properties in both animals and humans [1,2,3]. However, single plant extracts often fail to achieve the expected application objectives in commercial animal production owing to their limited biological functions. In recent years, a growing number of studies have demonstrated that compound plant extracts are more effective, attributed to their stronger and broader bioactivities. Mixed extracts derived from three to nine plant species have been reported to improve growth performance and carcass weight in pigs [4], reduce postprandial glycemia and attenuate diet-induced metabolic disorders [5], and inhibit inflammatory responses in rats [6]. On the other hand, emerging evidence indicates that certain traditional Chinese medicine prescriptions can improve health status and growth performance in farm animals, including weaned piglets, broilers, and beef cattle [7,8,9,10]. These findings suggest that combined plant extracts have gained increasing attention. Moreover, recently a few studies have further explored the molecular regulatory mechanisms of specific plant-derived extracts in humans and animals, involving the gut microbiota-immune axis [11,12], as well as inflammatory and antioxidant-associated signaling pathways [13,14].

Despite this progress, systematic strategies for plant selection, combination, and determination of optimal mixing ratios have rarely been reported. In our previous work, a four-plant formulation was developed based on comparative evaluation of the antibacterial and antioxidant activities of 17 individual plant extracts. This formulation was demonstrated to promote growth and alleviate diarrhea in mice [15] and to improve the health and growth performance while replacing dietary antibiotics in weaned piglets [16]. Notably, the observed efficacy was not detected in any of the individual constituent extracts [17], indicating both the feasibility of selecting plant materials based on in vitro bioactivities and the necessity of formulating multi-plant composite extracts. However, the application of this formulation has been limited by its high cost, which is primarily attributable to the relatively high dosage required in animal feed due to its moderate bioactivities.

Therefore, the present study aimed to develop new composite plant extracts with multiple bioactivities as alternatives to dietary antibiotics for application in livestock and poultry breeding. To achieve this objective, medicinal plants reported to possess stronger and more comprehensive biological effects than those used in our previous study were re-screened. In particular, we extended the range of in vitro bioactivity evaluation indexes, introduced an orthogonal experimental design for rational formulation optimization, and further validated the safety and efficacy of the developed composites in promoting health and improving growth performance in mice and piglets.

## 2. Materials and Methods

### 2.1. Preparation of Single Plant Extracts

A total of 23 plant materials, with prices listed in Table S1, were procured from the Pharmacy of Zhejiang Chinese Medical University (Hangzhou, China). The plant materials were thoroughly washed with water, oven-dried at 70 °C for 48 h, and ground into powder using a mechanical grinder. Each powdered material was extracted with 20 volumes of double-distilled water (ddH_2_O) in a heating water bath at 80 °C for 6 h, followed by filtration through 0.22 μm non-sterile needle filters to remove solid residues. The resulting filtrates were concentrated in an oven at 60 °C to a final concentration equivalent to 0.5 g of raw material per milliliter and stored at −20 °C until further analysis.

### 2.2. Assessment of Bioactivities In Vitro

#### 2.2.1. Antibacterial Assay

The swine-derived pathogenic *Escherichia coli* reference strain C83907 was obtained from the China Institute of Veterinary Drug Control (Hangzhou, China). Antibacterial activity was evaluated using the broth microdilution method as described by Shafiei et al. [18], with slight modifications. Briefly, bacterial inocula were prepared by culturing the strain in sterile Mueller–Hinton broth (MHB) at 37 °C for 16 h. The culture was subsequently serially diluted with MHB, and the inoculum concentration was adjusted to approximately 1 × 10^8^ CFU/mL using the serial dilution plating method. To determine the inhibitory effects of plant extracts against *E. coli*, 100 μL of extract and 40 μL of bacterial suspension were added to 4 mL of sterile MHB. Subsequently, 100 μL of the mixture was transferred into a 96-well plate and incubated at 37 °C with shaking at 210 rpm for 24 h. Sterile ddH_2_O was used in place of plant extracts as the control. All treatments were performed in triplicate. The optical density (OD) values of the cultures were measured at 600 nm using a spectrophotometer (BioTek Incorporated, Winooski, VT, USA) at 0 h (OD_0h_) and 24 h (OD_24h_) of incubation. The antibacterial rate was calculated using the following formula:

$$\mathrm{Antibaterial}\;\mathrm{rate}\%=\frac{\mathrm{Control}\;\left({\mathrm{OD}}_{24h}-{\mathrm{OD}}_{0h}\right)-\mathrm{Extract}\left({\mathrm{OD}}_{24h}-{\mathrm{OD}}_{0h}\right)}{\mathrm{Control}\left({\mathrm{OD}}_{24h}-{\mathrm{OD}}_{0h}\right)}\times 100\%$$

#### 2.2.2. Anti-Inflammatory Assay

The anti-inflammatory properties of plant extracts were evaluated by measuring their inhibitory effects on 5-lipoxygenase (5-LOX) activity, according to the method described by Trouillas et al. [19], with slight modifications. Briefly, a 5.4% linoleic acid solution was prepared in 0.2 mol/L boric acid buffer containing 0.5% Tween-20 and used as the substrate for 5-LOX. Each extract (1 μL) was added to 20 μL of 5-LOX solution diluted 50-fold with ddH_2_O and incubated at 30 °C for 30 min. Subsequently, 30 μL of substrate solution was added, followed by further incubation at 30 °C for 3 min. The reaction was terminated by the addition of 500 μL of dehydrated alcohol. The test solution was prepared by adding an additional 500 μL of ddH_2_O to the reaction mixture. Aliquots of 200 μL were transferred to a 96-well plate, and the absorbance (OD_a_) was measured at 234 nm using a spectrophotometer.

The control groups included ddH_2_O in place of plant extracts (OD_b_), 5-LOX solution alone (OD_c_), and 500 μL of dehydrated alcohol added prior to substrate addition with extracts omitted (OD_d_). All treatments were performed in triplicate. The anti-inflammatory rate was calculated using the following formula:

$$Anti\text{-}inflammatory\;rate\%=\frac{{OD}_{a}-{OD}_{c}}{{OD}_{d}-{OD}_{b}}\times 100\%$$

#### 2.2.3. Antioxidant Assay

The antioxidant properties of plant extracts were evaluated based on their ability to scavenge the 2,2-diphenyl-1-picrylhydrazine (DPPH) free radical, according to the method described by Aluko et al. [20], with slight modifications. Briefly, a 0.5 mg/mL DPPH solution was prepared using dehydrated alcohol. Plant extracts at concentrations of 100, 50, 25, 12.5, and 6.25 mg/mL were tested. Mixtures containing 100 μL of extract and an equal volume of DPPH solution were incubated in a 96-well plate at 37 °C for 30 min. The absorbance of each mixture (OD_extract_) was then measured at 516 nm using a spectrophotometer.

Sterile ddH_2_O was used in place of plant extracts as the control (OD_control_), and dehydrated alcohol was used in place of the DPPH solution as the blank (OD_blank_). All treatments were performed in triplicate. The antioxidant rate was calculated using the following formula:

$$Antioxidant{\;\mathrm{IC}}_{50}=\frac{{OD}_{control}-\left({OD}_{extract}-{OD}_{blank}\right)}{{OD}_{blank}}$$

The concentration of each extract required to scavenge 50% of DPPH free radicals was defined as the antioxidant IC_50_ value.

#### 2.2.4. Preparation of Candidate Composite Extracts by Orthogonal Design

An L_16_(4^5^) orthogonal experimental design was employed to develop candidate composite plant extracts. As shown in Table S2, five plant materials were defined as experimental factors, each with four usage concentrations as levels based on the Pharmacopoeia of the People’s Republic of China (2015) [21], resulting in 16 combinations listed in Table S3. The five plant materials were weighed according to the specified ratios, thoroughly mixed, and extracted using the extraction method described above. The antibacterial rate, anti-inflammatory rate, and antioxidant IC_50_ values of each extract were determined. Based on range and variance analyses, three candidate composite extracts, designated C1, C2, and C3, were selected. Their antibacterial, anti-inflammatory, and antioxidant activities were further validated using the corresponding methods described above.

### 2.3. Determination and Analysis of Total Polysaccharide Contents in Extracts

The total polysaccharide contents (TPC) of *Artemisia annua* (AA), *Cinnamomum cassia presl* (CCP), *Magnolia officinalis cortex* (MOC), *Punica granatum* L. *pericarpium* (PGP), *Spatholobi suberectus Dunn caulis* (SSC), and the three candidate composite extracts were determined according to the method described by Hu et al. [22], with slight modifications. Briefly, four volumes of 95% ethanol were added to the concentrated extract solutions, followed by incubation at room temperature for 8 h. The mixtures were then centrifuged at 1500× *g* for 20 min. The resulting precipitates were washed sequentially with acetone and ether and subsequently dissolved in ddH_2_O. Finally, the absorbance of the test solutions was measured at 490 nm. All treatments were performed in triplicate.

The theoretical TPC values of C1, C2, and C3 were calculated using the following formula:

$$Theoretical\;TPC\%=(\mathrm{AA}\%\times {\mathrm{TPC}}_{\mathrm{AA}}\%+\mathrm{CCP}\%\times {\mathrm{TPC}}_{\mathrm{CCP}}\%+\mathrm{MOC}\%\times {\mathrm{TPC}}_{\mathrm{MOC}}\%+\;\mathrm{PGP}\%\times {\mathrm{TPC}}_{\mathrm{PGP}}\%+\mathrm{SSC}\%\times {\mathrm{TPC}}_{\mathrm{SSC}}\%)/100$$

AA%, CCP%, MOC%, PGP%, and SSC% represent the respective proportions of each plant material.

TPC_AA_, TPC_CCP_, TPC_MOC_, TPC_PGP_, and TPC_SSC_ represent the measured TPC values of AA, CCP, MOC, PGP, and SSC, respectively.

### 2.4. Animal Feeding Experiments

#### 2.4.1. Mice Feeding Experiments

Female BALB/c mice weighing approximately 13–15 g were used in this study. All mice had *ad libitum* access to food and water and were housed in plastic cages under controlled environmental conditions, including a constant temperature of 22 ± 2 °C, relative humidity of 50 ± 10%, and a 12 h light/dark cycle.

Experiment 1: To evaluate the safety of C1, C2, and C3, a sub-acute toxicity test was conducted in accordance with the National Food Safety Standard for Acute Oral Toxicity Tests of China (GB 15193.3-2014) [23]. A total of 78 mice were randomly assigned to four groups, including one control group and three experimental groups (C1, C2, and C3). Control mice were orally administered 0.2 mL of ddH_2_O. For the C1, C2, and C3 groups, four dosage levels were included, with six mice per treatment. Each group received oral gavage of 0.2 mL of extract at doses of 125, 250, 500, or 1000 mg/kg, respectively. The selection of these doses referred to our previous report [17]. All mice were treated once daily in the morning for 21 consecutive days.

At the end of the experiment, mice were fasted for 12 h and weighed. Whole blood samples were collected from the retrobulbar intraorbital capillary plexus for the determination of hematological parameters, including red blood cell (RBC), white blood cell (WBC), and platelet (PLT) counts, as well as hemoglobin (HGB) content. Mice were then euthanized by cervical dislocation, and the abdominal cavity was opened to excise visceral organs, including the heart, liver, spleen, kidney, and thymus, which were subsequently weighed. Visceral indexes were calculated as percentages of live body weight.

Experiment 2: To evaluate the efficacy of the composite extracts, 120 mice were allocated into four treatment groups based on body weight. Each treatment consisted of six cages, with five mice per cage. The control (CON) group was orally administered ddH_2_O, whereas the C1, C2, and C3 groups received 250 mg/kg C1, 125 mg/kg C2, and 75 mg/kg C3, respectively. And the doses were set based on the results of experiment 1. The experimental period lasted for 21 days. Individual body weights and feed intake per cage were recorded. Growth performance parameters, including body weight gain, were calculated on a per-mouse basis, whereas feed intake and feed-to-gain ratio were calculated on a per-cage basis.

At the end of the feeding experiment, six mice from each treatment group (CON, C1, and C2) were randomly selected and euthanized, and colonic contents were collected immediately. The V3–V4 region of the 16S rRNA gene was sequenced using the Illumina MiSeq platform (Novogene, Beijing, China). Obtained sequences with ≥97% similarity were assigned to the same operational taxonomic units (OTUs). Observed species and α-diversity indexes (Simpson index, Shannon index, Chao1, and ACE) were calculated using QIIME2 (version 2025.7) software. The composition of dominant microbial communities were compared.

Experiment 3: This experiment was conducted to verify whether C1 and C2 could alleviate injuries in a lipopolysaccharide (LPS)-induced mouse model that mimics weaning stress–induced conditions in piglets. A total of 24 mice were randomly and equally assigned to four groups. Mice in the Blank and LPS groups were orally administered ddH_2_O, whereas mice in the LPS + C1 and LPS + C2 groups received 250 mg/kg C1 and 125 mg/kg C2, respectively. On day 20, mice in the LPS, LPS + C1, and LPS + C2 groups were intraperitoneally injected with LPS at a dose of 10 mg/kg body weight, while mice in the Blank group received an equal volume of normal saline.

All mice were euthanized by cervical dislocation 24 h after LPS injection. The abdominal cavity was opened, and gastrointestinal tissues were collected. A segment of mid-jejunum was excised and fixed in 10% formalin for morphological analysis using the hematoxylin–eosin (HE) staining method. Villus height (VH) and crypt depth (CD) were measured using an OLYMPUS BX20 light microscope (Tokyo, Japan) coupled with an Image-Pro Plus analysis system (Media Cybernetics, Rockville, MD, USA). Mean values were calculated from measurements of 10 individual villi and crypts per specimen.

The remaining mid-jejunal samples were used to determine the mRNA expression levels of inflammation-related genes by real-time quantitative polymerase chain reaction (RT-qPCR). The target genes and primer sequences are listed in Table 1. Total RNA was extracted using TRIzol reagent, and complementary DNA (cDNA) was synthesized using a ReverTra Ace qPCR RT kit (Toyobo, Osaka, Japan). Glyceraldehyde-3-phosphate dehydrogenase (GAPDH) was used as the reference gene. RT-qPCR was performed using a SYBR Green Real-Time PCR Master Mix (Toyobo) on an ABI StepOne Plus PCR system (Applied Biosystems, Foster City, CA, USA). Each sample was analyzed in triplicate, and the relative quantification (RQ) values of mRNA expression were calculated using the 2^−ΔΔCt^ method.

Liver samples were immediately snap-frozen in liquid nitrogen and stored at −80 °C until analysis. Antioxidant indexes, including superoxide dismutase (SOD) activity, glutathione peroxidase (GSH-Px) activity, total antioxidant capacity (T-AOC), and malondialdehyde (MDA) content, were determined using commercial assay kits (Nanjing Jiancheng Bioengineering Institute, China), following the manufacturer’s instructions.

#### 2.4.2. Piglets Feeding Experiment

A total of 72 healthy “Duroc × Landrace × Yorkshire” piglets were randomly assigned to two groups based on initial body weight and sex, with six pens per group and six piglets per pen. The control group was fed a basal diet supplemented with antibiotics (100 mg/kg olaquindox and 20 mg/kg enramycin), whereas the experimental group received the basal diet supplemented with 200 mg/kg C2 extract in powder form. The supplementation level of C2 was determined from mouse studies using dose extrapolation. The dietary composition and nutrient levels are presented in Table S4. All piglets had *ad libitum* access to feed and water. The feeding trial lasted for 28 days.

During the experimental period, feed intake was recorded on a pen basis, and diarrhea incidence was monitored daily for each piglet. Average daily gain (ADG), average daily feed intake (ADFI), and feed-to-gain ratio were calculated based on initial body weight, final body weight, and total feed consumption per pen. Diarrhea was assessed using a four-grade fecal consistency scoring system, as described by Byun et al. [24]. Briefly, fresh feces were scored as follows: 0 = solid, 1 = semisolid, 2 = semiliquid, and 3 = liquid. Scores of 2 and 3 were defined as diarrhea. The diarrhea rate was calculated using the following formula:

$$Diarrea\;rate\%=\frac{Number\;of\;diarrheic\;piglets\times Diarrhea\;days}{Total\;number\;of\;piglets\times Experimental\;days}\times 100\%$$

### 2.5. Statistical Analysis

All data are presented as means ± standard error (SE). Data from more than two groups were analyzed using one-way analysis of variance (ANOVA), followed by Duncan’s multiple comparison test, whereas comparisons between two groups were performed using independent-samples *t* tests. Statistical analyses were conducted using SPSS 22.0 software (IBM SPSS, New York, NY, USA). Orthogonal experimental design and range analyses of bioactivity data were performed using the Orthogonality Experiment Assistant 3.1 mini program. A value of *p* < 0.01 was considered extremely significant, and *p* < 0.05 was considered statistically significant.

## 3. Results

### 3.1. Antibacterial, Anti-Inflammatory, and Antioxidant Activities of Single Plant Extracts In Vitro

The antibacterial rates, anti-inflammatory rates, and antioxidant IC_50_ values of the 23 individual plant extracts are presented in Table 2. Among the 23 extracts, PGP exhibited the highest antibacterial rate (*p* < 0.01), followed by CCP and AA, which showed the second-highest antibacterial activity (*p* < 0.01); all three exceeded 55%. *Glycyrrhiza uralensis Fisch. radix et rhizome*, *Ginkgo billoba* L. *folium*, SSC, and MOC exhibited the third-highest antibacterial rates (*p* < 0.01), with no significant difference (*p* > 0.05) between MOC and *Acanthopanax senticosus radix et caulis*. The antibacterial rates of the remaining extracts were below 10%, except for *Filipendula palmate (pall.) Maxim*.

Seven extracts—PGP, MOC, SSC, *Rosmarinus officinalis* L., *Citrus limon* (L.) *Burm. f. fructus*, *Euphorbia helioscopia* L., and *Filipendula palmate (pall.) maxim.*—exhibited the highest anti-inflammatory rates (*p* < 0.01), followed by *Artemisia scoparia Thunb.*, *Epimedium brevicornu maxim. folium*, and CCP, which showed the second-highest activity (*p* < 0.01); all exceeded 65%. The remaining extracts showed anti-inflammatory rates ranging from 0.298% to 42.71%, with some exhibiting very significant differences (*p* < 0.01) among them.

The antioxidant IC_50_ values of the 23 extracts ranged from 0.085 to 188.9 mg/mL, with PGP exhibiting the lowest value. Most extracts demonstrated strong antioxidant activity, with IC_50_ values of 17 extracts below 30.0 mg/mL and no significant differences observed among them (*p* > 0.05). The remaining extracts exhibited IC_50_ values ranging from 37.75 to 188.9 mg/mL, with very significant differences (*p* < 0.01) among some of them.

Based on the combined evaluation of antibacterial, anti-inflammatory, and antioxidant activities, together with comparative cost considerations shown in Table S1, five plant materials—AA, CCP, MOC, PGP, and SSC—were selected as constituents for the formulation of candidate composite extracts.

### 3.2. Formulation of Candidate Composite Plant Extracts

**Figure 1:**
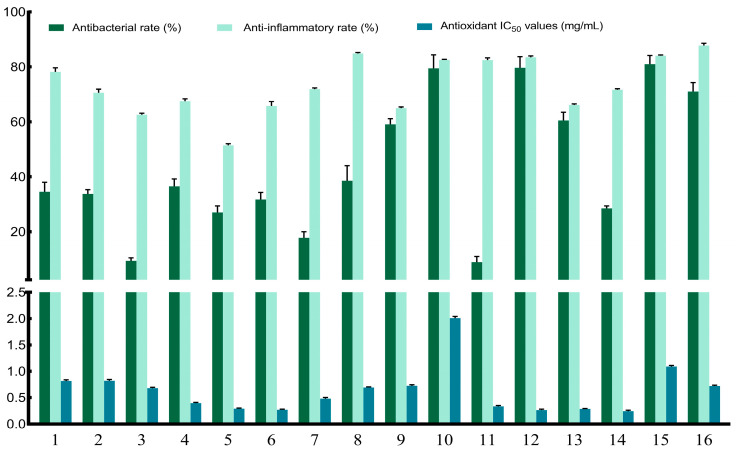
Bioactivities of 16 combinations generated by orthogonal design. Combinations 1–16 represent extracts prepared from the different raw plant material levels of *Artemisia annua* (AA), *Cinnamomum cassia presl* (CCP), *Magnolia officinalis cortex* (MOC), *Punica granatum* L. *pericarpium* (PGP), and *Spatholobi suberectus Dunn caulis* (SSC), respectively, which are listed in Table S3. Bars represent mean ± SE (*n* = 3).

Based on the range analysis results (Table S5) of the multi-bioactivity profiles (Figure 1) obtained from the 16 combinations generated by the orthogonal assay, three candidate composite extracts (C1, C2, and C3) were developed. The levels of AA, CCP, MOC, PGP, and SSC in C1 were 2, 4, 4, 1, and 4, respectively, corresponding to a formulation ratio of 6:9:7:1:13. Similarly, the levels of the five components in C2 were 1, 1, 4, 1, and 4, respectively, with a ratio of 4:1:7:1:13. For C3, the levels were 1, 1, 2, 4, and 4, respectively, corresponding to a ratio of 4:1:3:7:13. Theoretically, these three composite extracts were expected to exhibit optimal antibacterial (C1), anti-inflammatory (C2), and antioxidant (C3) activities, respectively, while retaining moderate levels of the other two bioactivities among all possible combinations.

### 3.3. In Vitro Bioactivity Verification of Extracts C1, C2, and C3

The detected bioactivities of C1, C2, and C3 are presented in Table 3. Extract C1 exhibited the highest antibacterial rate, with significant differences (*p* < 0.05) compared with C2 and C3, whereas no significant difference (*p* > 0.05) was observed between C2 and C3. Similarly, C2 showed the highest anti-inflammatory rate, which was significantly different (*p* < 0.01) from those of the other two extracts, while no significant difference (*p* > 0.05) was detected between C1 and C3. Regarding antioxidant activity, as indicated by IC_50_ values, the order was C2 < C1 < C3, with extremely significant differences (*p* < 0.01) observed between all pairwise comparisons.

### 3.4. Analytical Results of Total Polysaccharide Content in Extracts

The measured TPC values of AA, CCP, MOC, PGP, and SSC were 4.45%, 8.84%, 31.16%, 15.12%, and 9.17%, respectively. The measured and theoretical TPC values of C1 were 47.07% and 14.13%, those of C2 were 50.51% and 14.58%, and those of C3 were 45.40% and 11.69%. Notably, the measured TPC values of C1, C2, and C3 were 233.2%, 246.5%, and 288.3% higher than their corresponding theoretical values, respectively.

### 3.5. Safety Evaluation of Candidate Composite Extracts in Mice

In Experiment 1, hematological parameters and visceral indexes were used to assess whether the three candidate composite extracts exerted adverse effects on animal health within a defined dosage range. Throughout the feeding experiment, no mortality or abnormal clinical symptoms were observed in mice administered any of the extracts, even at the highest dose of 1000 mg/kg. As shown in Table 4, compared with the control group, no significant changes (*p* > 0.05) were observed in WBC, RBC, or PLT counts, or HGB concentrations at any dosage of C1, C2, or C3.

Regarding visceral indexes (Table 5), compared with the control group, the thymus index was significantly higher (*p* < 0.05) in mice treated with C1 at a dose of 1000 mg/kg, whereas no significant differences (*p* > 0.05) were observed in other indexes. At the remaining three doses of C1, all visceral indexes showed no significant differences (*p* > 0.05). No significant changes (*p* > 0.05) were detected in any visceral indexes at all tested doses of C2. In contrast, in the C3 group, the kidney index at 1000 mg/kg and the thymus indexes at 500 and 1000 mg/kg were significantly increased (*p* < 0.05). Overall, all composite extracts exhibited no adverse effects at doses below 500 mg/kg.

### 3.6. Efficacy of Candidate Composite Extracts in Mice

**Figure 2:**
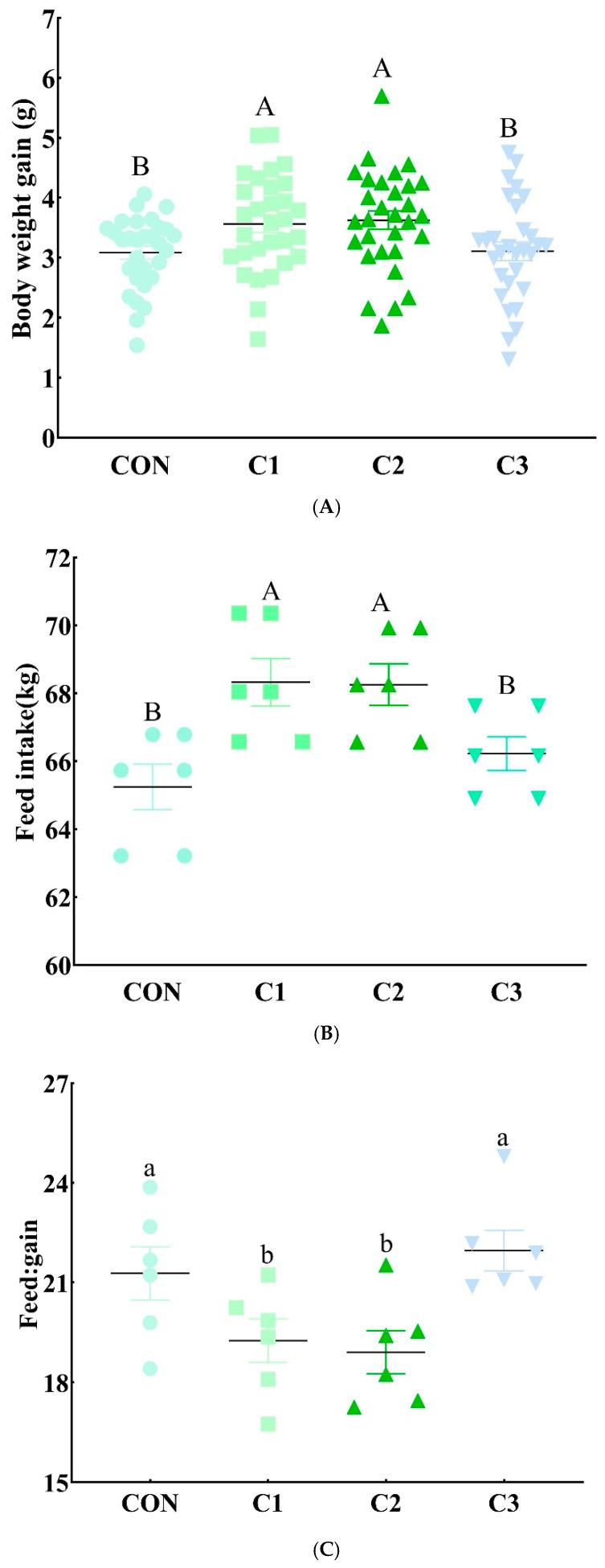
Effects of candidate composite extracts on growth performance in mice. (**A**) Body weight gain (*n* = 30); (**B**) feed intake (*n* = 6); (**C**) feed:gain ratio (*n* = 6). C1, C2, and C3 extracts were prepared from different raw plant material ratios of *Artemisia annua* (AA), *Cinnamomum cassia presl* (CCP), *Magnolia officinalis cortex* (MOC), *Punica granatum* L. *pericarpium* (PGP), and *Spatholobi suberectus Dunn caulis* (SSC), respectively. Different uppercase and lowercase letters on the data bars indicate extremely significant differences (*p* < 0.01) and significant differences (*p* < 0.05), respectively; identical letters or no letters indicate no significant differences (*p* > 0.05).

In Experiment 2, growth performance indexes and intestinal microbiota analyses were employed to evaluate the efficacy of the composite extracts in promoting animal growth and regulating gut microbial structure at appropriate doses. As shown in Figure 2, compared with the control group, both C1 and C2 significantly increased (*p* < 0.01) body weight gain and feed intake, and significantly decreased (*p* < 0.05) the feed-to-gain ratio, whereas C3 had no significant effects (*p* > 0.05). These results indicate that C1 and C2, but not C3, possess the potential to improve growth performance in animals. Therefore, C3 was excluded from subsequent experiments.

**Figure 3:**
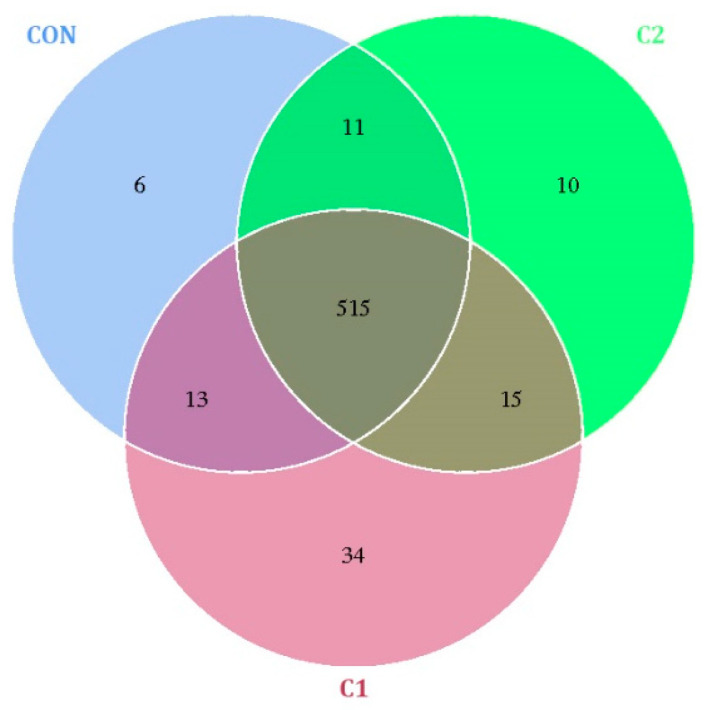
Venn diagram of intestinal microbiota OTUs in mice. C1 and C2, extracts prepared from the different raw plant material ratio of *Artemisia annua* (AA), *Cinnamomum cassia presl* (CCP), *Magnolia officinalis cortex* (MOC), *Punica granatum* L. *pericarpium* (PGP), and *Spatholobi suberectus Dunn caulis* (SSC), respectively. Each circle represents a group, and the numbers indicate the corresponding OTU counts. *n* = 6.

**Figure 4:**
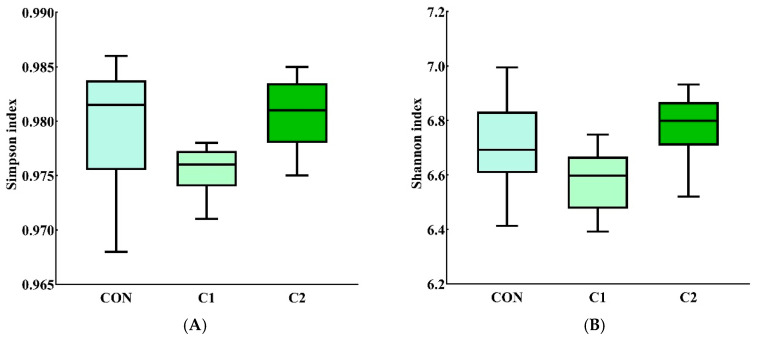

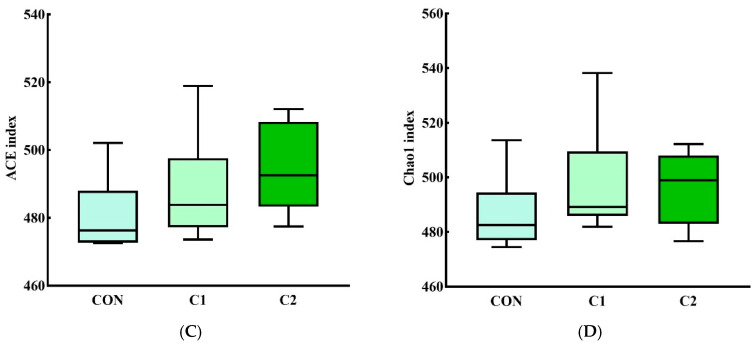
α-Diversity indexes of intestinal microbiota in mice. (**A**) Simpson; (**B**) Shannon; (**C**) ACE; (**D**) Chao1. C1 and C2, extracts prepared from the different raw plant material ratio of *Artemisia annua* (AA), *Cinnamomum cassia presl* (CCP), *Magnolia officinalis cortex* (MOC), *Punica granatum* L. *pericarpium* (PGP), and *Spatholobi suberectus Dunn caulis* (SSC), respectively. *n* = 6.

**Figure 5:**
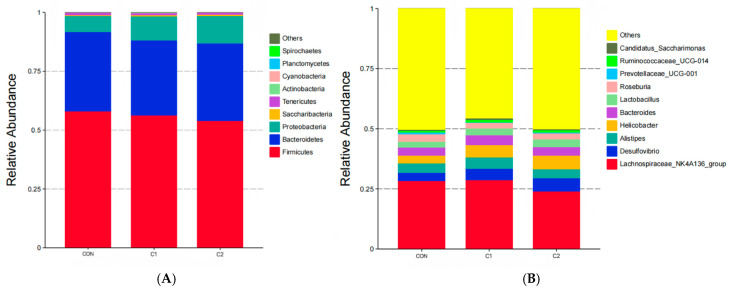
Relative abundance of the top 10 intestinal microbiota taxa in mice. (**A**) Phylum level; (**B**) genus level. C1 and C2, extracts prepared from the different raw plant material ratio of *Artemisia annua* (AA), *Cinnamomum cassia presl* (CCP), *Magnolia officinalis cortex* (MOC), *Punica granatum* L. *pericarpium* (PGP), and *Spatholobi suberectus Dunn caulis* (SSC), respectively.

The results of intestinal microbiota operational taxonomic unit (OTU) analysis are shown in Figure 3. Venn diagram analysis demonstrated that 515 OTUs were shared among all groups. The C1 group exhibited the highest number of unique OTUs, followed by C2, whereas the CON group exhibited the lowest number. Alpha-diversity analysis (Figure 4) revealed no significant differences (*p* > 0.05) among the groups. Microbial composition analysis (Figure 5, Tables S6 and S7) showed that *Firmicutes*, *Bacteroidetes*, and *Proteobacteria* were the most abundant phyla. The relative abundance of *Proteobacteria* was significantly increased (*p* < 0.01) in the C1 and C2 groups compared with the CON group. Among the top 10 most abundant genera, the abundance of *Helicobacter* was significantly higher (*p* < 0.05) in the C1 and C2 groups than in the CON group.

### 3.7. Intestinal and Hepatic Protective Effects of C1 and C2 in LPS-Challenged Mice

**Figure 6:**
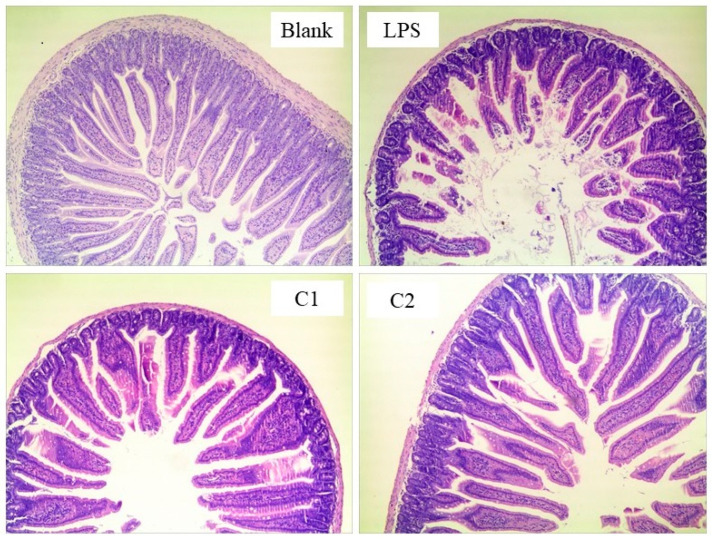
Hematoxylin and eosin (H&E)-stained intestinal morphology sections in mice (50×). C1 and C2 extracts were prepared from different raw plant material ratios of *Artemisia annua* (AA), *Cinnamomum cassia presl* (CCP), *Magnolia officinalis cortex* (MOC), *Punica granatum* L. *pericarpium* (PGP), and *Spatholobi suberectus Dunn caulis* (SSC), respectively.

In Experiment 3, jejunal morphological results are presented in Figure 6 and Table 6. Compared with the Blank group, lipopolysaccharide (LPS) injection caused marked damage to the small intestinal structure, characterized by villus fragmentation, lodging, and disordered arrangement, accompanied by a significant decrease (*p* < 0.01) in VH and the VH/CD ratio, and a significant increase (*p* < 0.01) in CD. Compared with the LPS group, both C1 and C2 markedly restored villus morphology, resulting in more intact and regularly arranged villi, and significantly improved (*p* < 0.01) VH, CD, and VH/CD values. However, the VH/CD ratio in the C1 group remained significantly lower (*p* < 0.01) than that of the Blank group.

**Figure 7:**
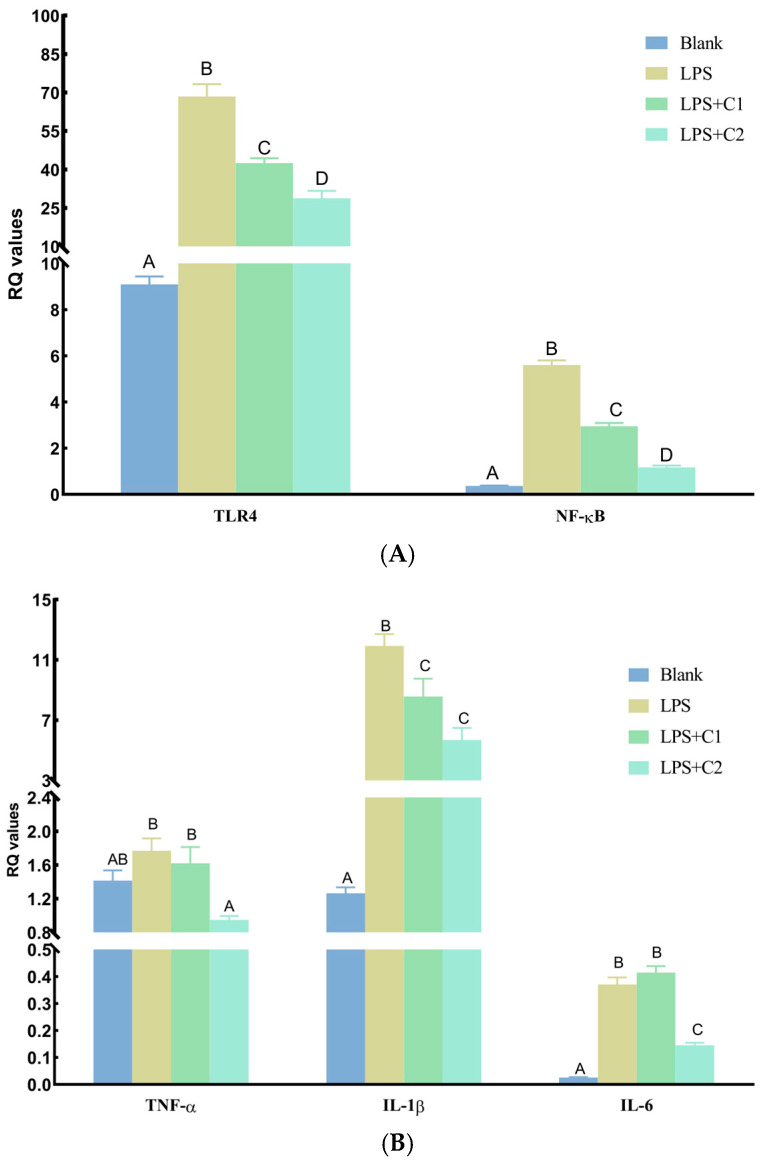
mRNA expression levels of intestinal inflammation-related genes in mice. (**A**) TLR4 and NF-κB; (**B**) TNF-α, IL-1β, and IL-6. C1 and C2 extracts were prepared from different raw plant material ratios of *Artemisia annua* (AA), *Cinnamomum cassia presl* (CCP), *Magnolia officinalis cortex* (MOC), *Punica granatum* L. *pericarpium* (PGP), and *Spatholobi suberectus Dunn caulis* (SSC), respectively. Bars represent mean ± SE (*n* = 6). Different uppercase letters on the data bars indicate extremely significant differences (*p* < 0.01); identical letters or no letters indicate no significant differences (*p* > 0.05).

The mRNA expression levels of intestinal inflammation-related genes are shown in Figure 7. Compared with the Blank group, LPS administration significantly increased (*p* < 0.01) the RQ values of all inflammatory-related genes except tumor necrosis factor-α (*TNF-α*). Compared with the LPS group, C1 significantly decreased (*p* < 0.01) the RQ values of toll-like receptor 4 (*TLR4*), nuclear factor kappa B (*NF-κB*), and interleukin-1β (*IL-1β*), but had no significant effects (*p* > 0.05) on *TNF-α* or interleukin-6 (*IL-6*). In contrast, C2 significantly decreased (*p* < 0.01) the RQ values of all tested genes. Furthermore, except for *IL-1β*, the RQ values of all genes were significantly lower (*p* < 0.01) in the C2 group than in the C1 group, although the expression levels of *TLR4*, *NF-κB*, *IL-1β*, and *IL-6* in both C1 and C2 groups remained significantly higher (*p* < 0.01) than those in the Blank group.

**Figure 8:**
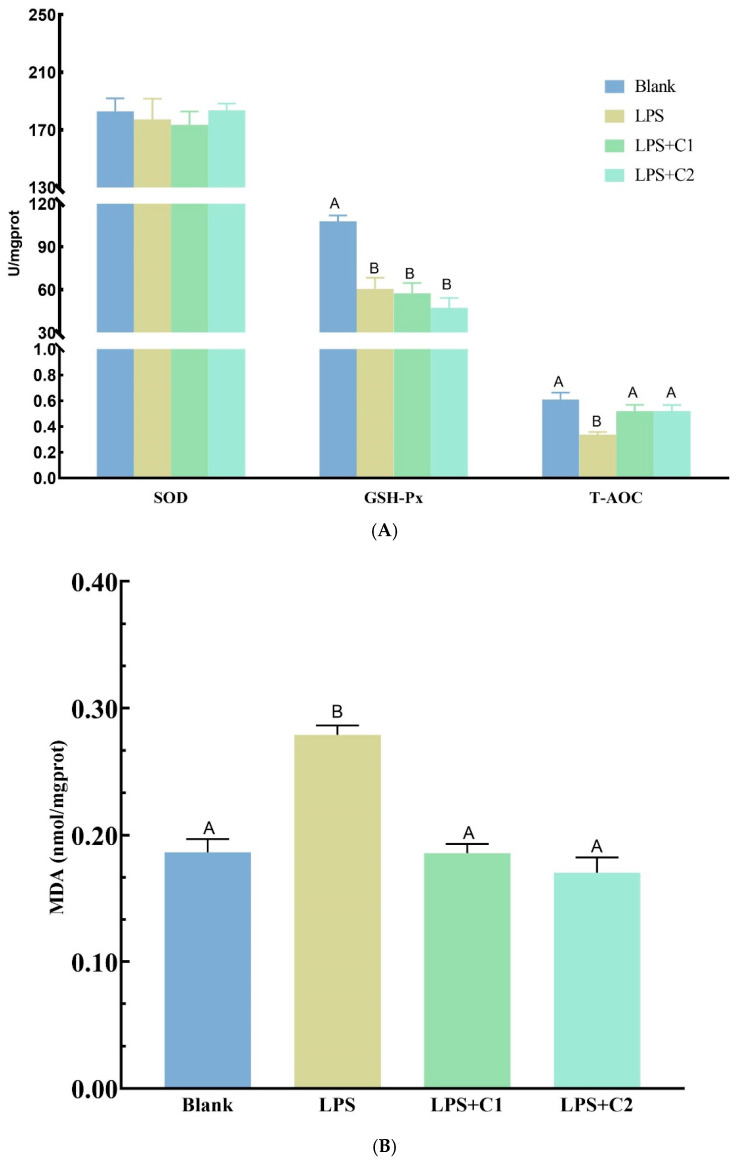
Hepatic antioxidant parameters in mice. (**A**) SOD, GSH-Px, and T-AOC; (**B**) MDA. C1 and C2 extracts were prepared from different raw plant material ratios of *Artemisia annua* (AA), *Cinnamomum cassia presl* (CCP), *Magnolia officinalis cortex* (MOC), *Punica granatum* L. *pericarpium* (PGP), and *Spatholobi suberectus Dunn caulis* (SSC), respectively. Bars represent mean ± SE (*n* = 6). Different uppercase letters on the data bars indicate extremely significant differences (*p* < 0.01); identical letters or no letters indicate no significant differences (*p* > 0.05).

Hepatic antioxidant results are presented in Figure 8. Compared with the Blank group, LPS administration significantly decreased (*p* < 0.01) the activities of GSH-Px and T-AOC, significantly increased (*p* < 0.01) MDA content, and had no significant effect (*p* > 0.05) on SOD activity. Compared with the LPS group, both C1 and C2 significantly increased (*p* < 0.01) T-AOC activity and significantly decreased (*p* < 0.01) MDA content to levels comparable to those of the Blank group, but exerted no significant effects (*p* > 0.05) on GSH-Px activity.

Based on these results, C2 was selected as a substitute for dietary antibiotics in subsequent experiments conducted in target animals.

### 3.8. Evaluation of C2 as a Replacement for Dietary Antibiotics in Weaned Piglets

As shown in Table 7, compared with the antibiotic group, dietary supplementation with C2 resulted in no significant differences (*p* > 0.05) in ADG, ADFI, feed-to-gain ratio, or diarrhea rate. These findings preliminarily indicate that the C2 composite extract exhibits could serve as a potential alternative to dietary antibiotics in weaned piglets.

## 4. Discussion

Except for PGP, the remaining 22 plants investigated in this study were not included in our previous work [17]. Accumulating evidence has demonstrated the pronounced antioxidant and antibacterial activities of PGP extracts, with its aqueous extract, in particular, exhibiting remarkable efficacy in both antibacterial and antioxidant capacities [25,26]. Notably, this study is the first to report that PGP exhibited the strongest anti-inflammatory activity among the 23 plants evaluated. In contrast, CCP, as an edible and medicinal material, has been widely recognized for its antibacterial, anti-inflammatory, and other bioactivities [27]. In the present study, CCP exhibited higher antioxidant activity than the other plants examined. The multi-bioactivities of AA and SSC have been comprehensively reviewed previously [28,29]. MOC has been recognized as a promising functional feed additive in the poultry industry [30], which is consistent with the bioactivities evaluated in this study. However, few studies have simultaneously investigated and compared the diverse bioactivities of multiple plant extracts. Despite the probable differences in bioactive component profiles, this study provides a comparative multi-bioactivity assessment of single extracts under a unified evaluation framework, with several findings rarely reported previously.

In the present study, an orthogonal assay was innovatively introduced for the rational formulation of composite extracts, resulting in the development of three optimal candidate formulas with distinct multi-bioactive characteristics across all possible combinations. Their prominent in vitro bioactivities were subsequently verified through comparative analysis. The strong multi-bioactivities of these formulas were likely attributable to synergistic effects arising during the combined extraction process. Similarly, previous studies have reported that combinations of individual plant extracts can generate synergistic antibacterial effects in vitro [31,32]. The enrichment of multiple active ingredients obtained from different plants likely contributed to enhanced bioactivities than single agents [33,34]. Notably, our TPC analytical results for the composite extracts confirmed the presence of such interactions, and we further emphasized that synergistic effects leading to increased polysaccharide content among different plants occur as early as the co-extraction process. This is likely attributed to the more thorough disruption of plant cell walls. Polysaccharides are among the primary bioactive components of traditional Chinese medicines used for the treatment of various diseases [35,36], and plant-derived polysaccharides have been shown to exert diverse biological activities in both animals and humans [37,38]. Future studies should therefore focus on elucidating the mechanisms underlying these synergistic effects and clarifying polysaccharide structure–activity relationships.

Most medicinal plant-derived extracts, particularly aqueous extracts, are generally considered safe due to their edible properties. In the present study, compared with C1 and C2, C3 exhibited weaker biological effects in mice, highlighting the importance of an optimized formulation. Similarly, phytogenic concentrations have been found to exert distinct effects on the structure of gut microbiota [39]. Intestinal microbiota is closely associated with animal growth and health. Plant extracts can act as natural modulators of intestinal microbiota composition, with different plant species shaping distinct microbial communities. Several studies have reported that AA and PGP modulate intestinal microbiota composition and improve intestinal health in livestock animals [40,41,42]. Specifically, PGP extracts have been shown to enhance gut microbial richness and increase the abundance of *Lactobacillaceae* and *Proteobacteria* in mice [43,44]. Consistently, the present study demonstrated that the formulated plant extracts regulated intestinal microbial communities and improved growth-related parameters in mice. These findings suggest that combined extraction may exert beneficial effects on gut microbiota. However, the observed increase in *Helicobacter* abundance is typically associated with impaired intestinal health. Thus, no definitive conclusion can be drawn regarding the effects of the extracts on intestinal microbial health due to the simultaneous observation of these contradictory findings. More experimental evidence is still required in the future.

The LPS-challenged mouse model has been widely used to investigate the mechanisms and protective effects of functional feed additives against stress-induced damage. An increasing number of studies have demonstrated the anti-inflammatory and antioxidant potential of various plant extracts in LPS-stimulated animals [45,46,47]. In the present study, an LPS-induced murine injury model was successfully established. Consistent with previous reports, MOC, AA, and CCP exhibited intestinal anti-inflammatory and protective effects, partially through the inhibition of pro-inflammatory factor expression [48,49,50]. Antioxidant effects of AA aqueous extracts have also been observed in the liver of mutton sheep [51]. In this study, both C1 and C2 alleviated LPS-induced intestinal and hepatic damage in mice, with C2 exhibiting superior anti-inflammatory activity. The molecular mechanism underlying these effects appears to be mediated, at least in part, by scavenging pro-inflammatory factors and reactive oxygen species stimulated by LPS. Consistent results have also been reported in several studies investigating other plant extracts and herbal polysaccharides [34,52,53]. Furthermore, the potential of C2 as a dietary antibiotic substitute was preliminarily validated in weaned piglets, a period characterized by weaning-induced inflammatory and oxidative stress and historically linked to the highest antibiotic usage in the swine industry, indicating strong applicability in livestock production.

Although the present study provides insights into the in vitro multi-bioactivities and significant effects of the composite plant extracts in animals, several limitations should be acknowledged. The target animal experiment was relatively short, used only at a single dose, with no in-depth mechanistic, pharmacokinetic or toxicological validation. Moreover, the restricted experimental setting limited the generalizability of the findings. Future studies will extend the experimental period and conduct mechanistic and safety evaluations to enhance the reliability of the formulation.

## 5. Conclusions

In summary, the study developed a new composite extract using an orthogonal assay, after selecting five plant materials (AA, CCP, MOC, PGP, and SSC) with favorable in vitro antibacterial, anti-inflammatory, and antioxidant activities from 23 candidate plants. The formulation was demonstrated to be safe and capable of improving growth performance in mice by regulating intestinal microbial communities, alleviating intestinal inflammation, and enhancing hepatic antioxidant capacity. Furthermore, it shows potential as an effective alternative to feed antibiotics in piglets. These results provide a promising new agent and offer novel perspectives for the exploration and development of antibiotic alternatives.

## Figures and Tables

**Table 1:** Primer sequences used for real-time quantitative polymerase chain reaction (RT-qPCR) analysis.

Genes	Primer Sequence (5′ to 3′)	Accession No.
*TLR4*	Forward: GGCATGGCATGGCTTACACCA	NM_021297.2
	Reverse: GAGAGGCCAATTTTGTCTCCACA	
*NF-κB*	Forward: CACAGACCCAGGAGTGTTCACAGA	M61909
	Reverse: GAGACATGGACACACCCTGGTTCA	
*IL-1β*	Forward: CACAACTGTTCGTGAGCGCTCAA	NM_010554
	Reverse: GAGTTTTGGTGTTTCTGGCAACTCCT	
*TNF-α*	Forward: GACCCTCACACTCAGATCATCTTCT	NM_013693
	Reverse: GCTACGACGTGGGCTACAG	
*IL-6*	Forward: GTGGCCCAGTACCAATGCGTCA	NM_010559
	Reverse: GGGCTCTGCTATCCAAGGAGTG	
*GAPDH*	Forward: GAAGGTCGGTGTGAACGGATTTG	GU214026.1
	Reverse: CATGTAGACCATGTAGTTGAGGTCA	

*TLR4*, toll like receptor 4; *NF-κB*, nuclear factor kappa-B; *TNF-α*, tumor necrosis factor-alpha; *IL-1β*, interleukin-1beta; *IL-6*, interleukin-6; *GAPDH*, glyceraldehyde-3-phosphate dehydrogenase.

**Table 2:** Comparison of bioactivities among 23 single extracts.

Plant Sources	Bioactivities		
	Antibacterial Rate%	Anti-Inflammatory Rate%	Antioxidant IC_50_ (mg/mL)
*Acanthopanax senticosus radix et caulis*	24.67 ± 0.779 ^DE^	30.84 ± 0.667 ^D^	53.88 ± 3.255 ^BC^
*Achyranthes bidentata radix*	7.667 ± 0.882 ^FG^	6.021 ± 0.370 ^FG^	188.9 ± 22.94 ^A^
*Artemisia annua*	57.03 ± 6.961 ^B^	0.377 ± 0.096 ^G^	3.335 ± 0.625 ^DE^
*Artemisia scoparia Thunb.*	6.638 ± 1.043 ^FG^	64.91 ± 2.352 ^B^	1.515 ± 0.475 ^DE^
*Atractylodes macrocephala Koidz. rhizoma*	7.881 ± 0.944 ^FG^	5.286 ± 0.332 ^FG^	11.25 ± 0.335 ^DE^
*Bambusoideae folium*	6.222 ± 0.801 ^FG^	0.298 ± 0.145 ^G^	26.21 ± 0.870 ^CDE^
*Cinnamomum cassia presl*	61.83 ± 5.778 ^B^	61.84 ± 0.120 ^B^	10.80 ± 0.390 ^DE^
*Citrus limon* (L.) *Burm. f. fructus*	6.276 ± 0.573 ^FG^	82.31 ± 0.901 ^A^	22.17 ± 1.605 ^CDE^
*Cyperus rotundus* L. *fructus*	6.209 ± 0.832 ^FG^	10.69 ± 1.456 ^F^	37.75 ± 1.665 ^CD^
*Epimedium brevicornu maxim. folium*	5.992 ± 0.817 ^FG^	65.16 ± 1.854 ^B^	6.040 ± 0.700 ^DE^
*Euphorbia helioscopia* L.	7.861 ± 1.284 ^FG^	80.41 ± 1.042 ^A^	14.58 ± 1.495 ^DE^
*Filipendula palmate (pall.) maxim.*	15.64 ± 2.557 ^EF^	80.13 ± 2.674 ^A^	8.855 ± 0.335 ^DE^
*Fraxinus rhynchophylla Hance cortex*	9.904 ± 1.192 ^FG^	42.71 ± 2.516 ^C^	0.865 ± 0.155 ^E^
*Ginkgo billoba* L. *folium*	35.50 ± 3.527 ^C^	20.93 ± 2.603 ^E^	3.905 ± 1.035 ^DE^
*Glycyrrhiza uralensis Fisch. radix et rhizome*	35.95 ± 5.758 ^C^	9.630 ± 1.310 ^F^	82.11 ± 3.655 ^B^
*Gynostemma pentaphyllum (Thunb.) markino*	5.153 ± 0.218 ^FG^	29.58 ± 2.851 ^D^	1.225 ± 0.125 ^E^
*Houttuynia cordata Thunb.*	9.419 ± 0.20 ^FG^	0.661 ± 0.106 ^G^	5.137 ± 1.160 ^DE^
*Ligustrum lucidum Ait. fructus*	6.815 ± 0.788 ^FG^	17.73 ± 3.285 ^E^	2.820 ± 0.454 ^DE^
*Magnolia officinalis cortex*	26.37 ± 1.691 ^CD^	82.28 ± 0.874 ^A^	1.730 ± 0.190 ^DE^
*Punica granatum* L. *pericarpium*	75.67 ± 3.383 ^A^	86.48 ± 1.362 ^A^	0.085 ± 0.005 ^E^
*Psoralea corylifolia* L. *fructus*	5.194 ± 0.350 ^FG^	0.593 ± 0.066 ^G^	182.1 ± 26.42 ^A^
*Rosmarinus officinalis* L.	0.800 ± 0.120 ^G^	81.73 ± 0.241 ^A^	50.70 ± 0.075 ^BC^
*Spatholobus suberectus Dunn caulis*	28.44 ± 3.021 ^CD^	83.35 ± 0.897 ^A^	2.043 ± 0.985 ^DE^

Values are mean ± SE (*n* = 3). Different capital letter superscripts in the same column indicate very significant differences (*p* < 0.01), identical letters indicate no significant differences (*p* > 0.05).

**Table 3:** Validation of bioactivities of candidate composite extracts.

Extracts	Bioactivities		
	Antibacterial Rate%	Anti-Inflammatory Rate%	Antioxidant IC_50_ (mg/mL)
C1	87.83 ± 0.628 ^a^	88.17 ± 0.546 ^B^	0.314 ± 0.028 ^B^
C2	82.44 ± 1.518 ^b^	92.65 ± 0.871 ^A^	0.396 ± 0.023 ^A^
C3	81.85 ± 1.089 ^b^	88.80 ± 0.286 ^B^	0.222 ± 0.011 ^C^

C1, C2, and C3, extracts prepared from the different raw plant material ratio of *Artemisia annua* (AA), *Cinnamomum cassia presl* (CCP), *Magnolia officinalis cortex* (MOC), *Punica granatum* L. *pericarpium* (PGP), and *Spatholobi suberectus Dunn caulis* (SSC), respectively. Values are mean ± SE (*n* = 3). Different lowercase letter superscripts in the same column indicate significant differences (*p* < 0.05), whereas different capital lowercase letter superscripts indicate very significant differences (*p* < 0.01), identical letters indicate no significant differences (*p* > 0.05).

**Table 4:** Effects of candidate composite extracts on blood cell parameters in mice.

Treatments	Dose (mg/kg)	Blood Cell Parameters			
		WBC (10^9^/L)	RBC (10^12^/L)	PLT (10^9^/L)	HGB (g/L)
Control		4.720 ± 0.550	11.86 ± 0.162	669.0 ± 37.59	188.60 ± 2.337
C1	125	6.400 ± 0.603	11.72 ± 0.112	494.83 ± 44.53	185.67 ± 1.667
	250	8.567 ± 0.761	11.53 ± 0.150	563.17 ± 30.83	180.33 ± 1.961
	500	5.633 ± 0.565	11.08 ± 0.513	492.83 ± 17.29	181.50 ± 2.643
	1000	4.350 ± 0.226	11.04 ± 0.133	534.00 ± 51.17	169.50 ± 3.442
C2	125	4.533 ± 0.683	11.72 ± 0.118	624.67 ± 54.09	183.83 ± 1.956
	250	6.517 ± 0.344	11.29 ± 0.079	535.17 ± 42.65	173.67 ± 2.333
	500	5.917 ± 0.697	11.70 ± 0.307	555.17 ± 72.04	182.17 ± 5.192
	1000	3.917 ± 0.326	11.18 ± 0.197	530.83 ± 36.11	174.33 ± 3.221
C3	125	4.750 ± 0.898	11.13 ± 0.172	574.33 ± 74.42	176.67 ± 2.512
	250	6.083 ± 0.687	10.90 ± 0.339	773.00 ± 137.65	168.50 ± 5.500
	500	3.383 ± 0.749	11.59 ± 0.236	770.33 ± 76.28	179.67 ± 2.871
	1000	6.420 ± 0.805	10.48 ± 0.361	561.80 ± 42.48	163.80 ± 6.119

WBC, white blood cell; RBC, red blood cell; PLT, platelet; HGB, hemoglobin. C1, C2, and C3, extracts prepared from the different raw plant material ratio of *Artemisia annua* (AA), *Cinnamomum cassia presl* (CCP), *Magnolia officinalis cortex* (MOC), *Punica granatum* L. *pericarpium* (PGP), and *Spatholobi suberectus Dunn caulis* (SSC), respectively. Values are mean ± SE (*n* = 6). No letters in the same column indicate no significant differences (*p* > 0.05).

**Table 5:** Effects of candidate composite extracts on visceral indexes in mice.

Treatments	Dose (mg/kg)	Visceral Indexes%				
		Heart	Liver	Kidney	Spleen	Thymus
Control		0.420 ± 0.014	3.814 ± 0.309	1.088 ± 0.111 ^a^	0.311 ± 0.011	0.228 ± 0.013 ^a^
C1	125	0.385 ± 0.078	3.727 ± 0.281	1.134 ± 0.026 ^a^	0.294 ± 0.010	0.265 ± 0.031 ^a^
	250	0.458 ± 0.020	3.006 ± 0.606	1.070 ± 0.039 ^a^	0.330 ± 0.023	0.257 ± 0.014 ^a^
	500	0.442 ± 0.025	3.718 ± 0.069	1.115 ± 0.020 ^a^	0.320 ± 0.014	0.254 ± 0.014 ^a^
	1000	0.444 ± 0.025	3.848 ± 0.136	1.164 ± 0.027 ^a^	0.332 ± 0.012	0.316 ± 0.023 ^b^
C2	125	0.420 ± 0.012	3.556 ± 0.052	0.985 ± 0.199 ^a^	0.316 ± 0.009	0.233 ± 0.018 ^a^
	250	0.471 ± 0.023	3.755 ± 0.074	1.126 ± 0.039 ^a^	0.294 ± 0.011	0.227 ± 0.023 ^a^
	500	0.482 ± 0.040	3.750 ± 0.047	1.183 ± 0.026 ^a^	0.351 ± 0.009	0.278 ± 0.018 ^a^
	1000	0.536 ± 0.049	4.130 ± 0.119	1.243 ± 0.037 ^a^	0.345 ± 0.020	0.235 ± 0.025 ^a^
C3	125	0.326 ± 0.065	3.754 ± 0.088	1.202 ± 0.020 ^a^	0.317 ± 0.013	0.178 ± 0.040 ^a^
	250	0.467 ± 0.035	4.043 ± 0.265	1.227 ± 0.030 ^a^	0.431 ± 0.053	0.234 ± 0.454 ^a^
	500	0.479 ± 0.054	3.794 ± 0.158	1.183 ± 0.029 ^a^	0.289 ± 0.017	0.302 ± 0.015 ^b^
	1000	0.491 ± 0.046	4.160 ± 0.177	1.402 ± 0.044 ^b^	0.345 ± 0.028	0.296 ± 0.014 ^b^

C1, C2, and C3, extracts prepared from the different raw plant material ratio of *Artemisia annua* (AA), *Cinnamomum cassia presl* (CCP), *Magnolia officinalis cortex* (MOC), *Punica granatum* L. *pericarpium* (PGP), and *Spatholobi suberectus Dunn caulis* (SSC), respectively. Values are mean ± SE (*n* = 6). Different lowercase letter superscripts in the same column indicate significant differences (*p* < 0.05), no letter or identical letters indicate no significant differences (*p* > 0.05).

**Table 6:** Effects of extracts C1 and C2 on intestinal mucosal morphology in LPS-challenged mice.

Items	Treatments			
	Blank	LPS	LPS + C1	LPS + C2
VH (µm)	474.23 ± 16.84 ^A^	331.00 ± 24.60 ^B^	447.31 ± 35.70 ^A^	462.49 ± 15.89 ^A^
CD (µm)	155.90 ± 8.950 ^B^	219.87 ± 12.96 ^A^	185.59 ± 9.393 ^B^	168.15 ± 6.424 ^B^
VH/CD	3.076 ± 0.147 ^A^	1.537 ± 0.151 ^C^	2.406 ± 0.1116 ^B^	2.769 ± 0.142 ^AB^

VH, Villus height; CD, Crypt depth. C1 and C2 extracts were prepared from different raw plant material ratios of Artemisia annua (AA), Cinnamomum cassia *presl* (CCP), *Magnolia officinalis cortex* (MOC), *Punica granatum* L. *pericarpium* (PGP), and *Spatholobi suberectus Dunn caulis* (SSC), respectively. Values are mean ± SE (*n* = 6). Different capital letter superscripts in the same row indicate very significant differences (*p* < 0.01); identical letters indicate no significant differences (*p* > 0.05).

**Table 7:** Effects of C2 on growth performance and diarrhea rate in weaned piglets.

Items	Treatments	
	Antibiotic	Extract C2
Initial body weight (kg)	7.70 ± 0.023	7.697 ± 0.030
Initial body weight (kg)	21.17 ± 0.715	21.14 ± 0.436
ADG (g/d)	480.9 ± 25.63	480.0 ± 38.57
ADFI (g/d)	612.8 ± 20.98	604.5 ± 15.70
Feed:gain	1.285 ± 0.128	1.262 ± 0.703
Diarrhea rate%	11.61 ± 1.346	9.127 ± 1.126

C2, composite plant extract from the raw material ratio of *Artemisia annua* (AA), *Cinnamomum cassia presl* (CCP), *Magnolia officinalis cortex* (MOC), *Punica granatum* L. *pericarpium* (PGP), and *Spatholobi suberectus Dunn caulis* (SSC) is 4:1:7:1:13, respectively. Values are mean ± SE (*n* = 6). No letters in the same row indicate no significant differences (*p* > 0.05).

## Data Availability

The original contributions presented in this study are included in the article/Supplementary Materials. Further inquiries can be directed to the corresponding authors.

## Supplementary Materials

The following supporting information can be downloaded at: https://www.mdpi.com/article/10.3390/antiox15040468/s1, Table S1. Plant sources and prices of the 23 single extracts; Table S2. Factors and levels used in the orthogonal experimental design; Table S3. Plant combinations generated using the orthogonal design; Table S4. Ingredient composition and nutrient levels of the basal diet (as-fed basis); Table S5. Results of range analysis of the bioactivities of extract combinations; Table S6. Effects of extracts C1 and C2 on the relative abundance (%) of dominant colonic microbial communities at the phylum level in mice; Table S7. Effects of extracts C1 and C2 on the relative abundance (%) of dominant colonic microbial communities at the genus level in mice.

## References

[1] Chen C., Chen L., Mao C., Jin L., Wu S., Zheng Y., Cui Z., Li Z., Zhang Y., Zhu S. (2024). Natural extracts for antibacterial applications. Small.

[2] Rodríguez-Yoldi M.J. (2021). Anti-inflammatory and antioxidant properties of plant extracts. Antioxidants.

[3] Muntean D., Vulpie S. (2023). Antioxidant and antibacterial activity of plant extracts. Antibiotics.

[4] Dávila-Ramírez J.L., Munguía-Acosta L.L., Morales-Coronado J.G., García-Salinas A.D., González-Ríos H., Celaya-Michel H., Sosa-Castañeda J., Sánchez-Villalba E., Anaya-Islas J., Barrera-Silva M.A. (2020). Addition of a mixture of plant extracts to diets for growing-finishing pigs on growth performance, blood metabolites, carcass traits, organ weight as a percentage of live weight, quality and sensorial analysis of meat. Animals.

[5] Jurgoński A., Billing-Marczak K., Jukiewicz J., Krotkiewski M. (2019). Formulation of a mixture of plant extracts for attenuating postprandial glycemia and diet-induced disorders in rats. Molecules.

[6] Seo T.S., Lee J.H., Kim D.W., Lee D.C. (2025). Functional evaluation of extracts from mixed natural plants (MENP) for oral health: Anti-inflammatory and alveolar bone protective effects in periodontitis model rats. Food Sci. Biotechnol..

[7] Gao J., Wang R., Liu J.X., Wang W.L., Chen Y., Cai W.T. (2022). Effects of novel microecologics combined with traditional Chinese medicine and probiotics on growth performance and health of broilers. Poult. Sci..

[8] Wang C.H., Chung K.T., Su L.Y., Wu W.J., Wang P.H., Lee M.C., Shen S.C., Wu C.H. (2024). Chinese herbal medicines as natural alternative products to antibiotics in weaned piglets through intestinal microbiota regulation. Int. J. Mol. Sci..

[9] Ding P., Wang X., Jiang S., Li M., He X., Peng Y. (2025). Effects of bacteria-enzyme co-fermented Chinese herbal medicine on growth performance, apparent nutrient digestibility, meat quality, and immune function in broilers. Front. Vet. Sci..

[10] Liu Q., Zhang X.Y., Li X.W., Li J.Y., Guo L., Zhong W. (2025). Effects of fermentation duration on nutritional value, probiotic components and growth performance in beef cattle fed fermented Chinese medicine residue. J. Appl. Anim. Res..

[11] Jiang C., Chen D., Li J., Yue X., Wang S., Cao G., Chi S., Zhang Y. (2026). Lycium barbarum Polysaccharides-enriched extract Ameliorate Rheumatoid Arthritis via Gut microbiota-mediated regulation of the Tfh/B cell axis. Phytomedicine.

[12] Jiang T., Zhu R., Guo X., Li J., Zhu X., Bao R., Chen J. (2026). Comprehensive evaluation of Ganoderma lucidum extracts: Digestion kinetics, gut microbiota modulation, and immunoregulatory mechanisms. Food Res. Int..

[13] Tu L., Zhu X., Peng M., Li H., Wang Y., Liu J., Yang X., Chen L., Yang Y., Li R. (2026). Magnolia officinalis Rehder & E. Wilson extract and its main component honokiol alleviate asthma by reducing respiratory inflammation through the TRPV1/NFAT/TSLP pathway. Phytomedicine.

[14] Baishan A., Dilimulati D., Aikebaier A., Paerhati Y., Qiu X., Yusufujiang N., Wusiman Y., Abudoureheman A., Zhou W. (2026). Multi-Target Cardioprotection from *Berberis kaschgarica* Extract in Zebrafish via AMPK Pathway Activation. Antioxidants.

[15] Tao X., Sun Y., Men X., Xu Z. (2020). A compound plant extract and its antibacterial and antioxidant properties in vitro and in vivo. 3 Biotech.

[16] Tao X., Sun Y., Men X., Deng B., Li Y., Xu Z. (2018). Effects of composite plant extracts replacing feed antibiotics on growth performance and serum indexes of weaned piglets. Chin. J. Anim. Nutr..

[17] Sun Y. (2012). The components, safety evaluation, and application study on mouse of the compound plant extracts. Master’s Thesis.

[18] Shafiei Z., Haji A.R.Z., Philip K., Thurairajah N. (2016). Antibacterial and anti-adherence effects of a plant extract mixture (PEM) and its individual constituent extracts (*Psidium* sp., *Mangifera* sp., and *Mentha* sp.) on single- and dual-species biofilms. Peer J..

[19] Trouillas P., Calliste C.A., Allais D.P., Simon A., Marfak A., Delage C., Duroux J.L. (2003). Antioxidant, anti-inflammatory and antiproliferative properties of sixteen water plant extracts used in the limousin countryside as herbal teas. Food Chem..

[20] Aluko R.E., Monu E. (2003). Functional and bioactive properties of quinoa seed protein hydrolysates. J. Food Sci..

[21] Chinese Pharmacopoeia Commission (2015). Pharmacopoeia of the People’s Republic of China.

[22] Hu M., Zhang H., Feng B., Liu K., Guo S. (2013). Extraction of polysaccharides from Fomes officinalis Ames and their antitumor activity. Exp. Ther. Med..

[23] (2025). National Food Safety Standard for Acute Oral Toxicity Test.

[24] Byun Y.J., Lee C.Y., Kim M.H., Jung D.Y., Han J.H., Jang I., Song Y.M., Park B.C. (2017). Effects of dietary supplementation of a lipid-coated zinc oxide product on the fecal consistency, growth, and morphology of the intestinal mucosa of weanling pigs. J. Anim. Sci. Technol..

[25] Abu-Niaaj L.F., Al-Daghistani H.I., Katampe I., Abu-Irmaileh B., Bustanji Y.K. (2024). Pomegranate peel: Bioactivities as antimicrobial and cytotoxicagents. Food Sci. Nutr..

[26] Sharayei P., Azarpazhooh E., Ramaswamy H.S. (2020). Effect of microencapsulation on antioxidant and antifungal properties of aqueous extract of pomegranate peel. J. Food Sci. Technol. Mysore.

[27] Zhang C., Fan L., Fan S., Wang J., Luo T., Tang Y., Chen Z., Yu L. (2019). *Cinnamomum cassia presl*: A review of its traditional uses, phytochemistry, pharmacology and toxicology. Molecules.

[28] Shaer N.A., Mohamed A.A., Schnug E. (2025). Potential of artemisia annua bioactives as antiviral agents against SARS-CoV-2 and other health complications. Pharmaceuticals.

[29] Pan Y.H., Luo X.M., Gong P.Y. (2023). *Spatholobi caulis*: A systematic review of its traditional uses, chemical constituents, biological activities and clinical applications. J. Ethnopharmacol..

[30] Li Z., Ali S., Behan A.A., Arain M.A., Buzdar J.A., Yuan H. (2025). Exploring Magnolia officinalis and their derivatives as a functional feed additive to modulate poultry health and performance: A-review. World’s Poult. Sci. J..

[31] Jeong J.Y., Jung I.G., Yum S.H., Hwang Y.J. (2023). In vitro synergistic inhibitory effects of plant extract combinations on bacterial growth of methicillin-resistant *Staphylococcus aureus*. Pharmaceuticals.

[32] Sitarek P., Merecz-Sadowska A., Kowalczyk T., Wieczfinska J., Zajdel R., Śliwiński T. (2020). Potential synergistic action of bioactive compounds from plant extracts against skin infecting microorganisms. Int. J. Mol. Sci..

[33] Üzer F.B., Helvacı N., Elmastaş M. (2025). Synergistic Phenolic Compounds in Medicinal Plant Extracts: Enhanced Furin Protease Inhibition via Solvent-Specific Extraction from *Lamiaceae* and *Asteraceae* Families. Molecules.

[34] Ahmed R.F., Rasheed D.M., Mowaad N.A., Elgohary R., Eltantawy E.H., Negm E.A., Farag M.A., Elshamy A.I. (2026). Synergistic wound healing mechanisms of *Heliotropium curassavicum* extracts via redox modulation, inflammation suppression, and tissue remodeling: Linking phytochemical diversity to antioxidant and anti-inflammatory effects. Inflammopharmacology.

[35] Song F., Mei Z., Zhang W. (2026). Advances in polysaccharides from traditional Chinese medicine for the treatment of central nervous system disorders: A comprehensive review. Int. J. Biol. Macromol..

[36] Wang J., Wu X., Chen J., Gao T., Zhang Y., Yu N. (2024). Traditional Chinese medicine polysaccharide in nano-drug delivery systems: Current progress and future perspectives. Biomed. Pharmacother..

[37] Wang X., Li X., Zhang L., Xie Z., Guo L., Gao W. (2025). Plant derived polysaccharides as prebiotic-like components: Natural sources of glycolipid regulators and substitutes. Food Chem..

[38] Wang H., Li H., Hou Y., Zhang P., Tan M. (2023). Plant polysaccharides: Sources, structures, and antidiabetic effects. Curr. Opin. Food Sci..

[39] Bajagai Y.S., Alsemgees J., Moore R.J., Van S.D. (2020). Phytogenic products, used as alternatives to antibiotic growth promoters, modify the intestinal microbiota derived from a range of production systems: An in vitro model. Appl. Microbiol. Biotechnol..

[40] Liu S., Li S., Lu S., Yang M., Liu M., Li J., Li S., Jian F. (2025). Effects of fermented *Artemisia annua* on the intestinal microbiota and metabolites of Hu lambs with naturally infected with *Eimeria* spp. Front. Cell. Infect. Microbiol..

[41] Xu P., Wang J., Chen P., Ding H., Wang X., Li S., Fan X., Zhou Z., Shi D., Li Z. (2024). Effects of pomegranate (*Punica granatum* L.) peel on the growth performance and intestinal microbiota of broilers challenged with *Escherichia coli*. Poult. Sci..

[42] Cui Y., Leng X., Zhao Y., Zhao Y., Wang Q. (2024). Effects of dietary *Artemisia annua* supplementation on growth performance, antioxidant capacity, immune function, and gut microbiota of geese. Poult. Sci..

[43] Lu X.Y., Han B., Deng X., Deng S.Y., Zhang Y.Y., Shen P.X., Hui T., Chen R.H., Li X., Zhang Y. (2020). Pomegranate peel extract ameliorates the severity of experimental autoimmune encephalomyelitis via modulation of gut microbiota. Gut Microbes.

[44] George N.S., Cheung L., Luthria D.L., Santin M., Dawson H.D., Bhagwat A.A., Smith A.D. (2019). Pomegranate peel extract alters the microbiome in mice and dysbiosis caused by *Citrobacter rodentium* infection. Food Sci. Nutr..

[45] Zhang C., Wang S., Han Y., Zheng A., Liu G., Meng K., Yang P., Chen Z. (2024). Effects of crude extract of Glycyrrhiza Radix and *Atractylodes macrocephala* on immune and antioxidant capacity of SPF white leghorn chickens in an oxidative stress model. Antioxidants.

[46] Wan Y., Fu Y., Wang F., Sinclair A.J., Li D. (2018). Protective effects of a lipid extract from hard-shelled mussel (*Mytilus coruscus*) on intestinal integrity after lipopolysaccharide challenge in mice. Nutrients.

[47] Song W., Li Y., Zhang X., Wang Z. (2019). Effects of *Blidingia* sp. extract on intestinal inflammation and microbiota composition in LPS-Challenged mice. Front. Physiol..

[48] Liu S., Yang D., Li W., Chen Q., Lu D., Xiong L., Wu J., Ao H., Huang L. (2024). Magnolia officinalis alcohol extract alleviates the intestinal injury induced by polygala tenuifolia through regulating the PI3K/AKT/NF-κB signaling pathway and intestinal flora. Drug Des. Dev. Ther..

[49] Song Z., Cheng K., Zhang L., Wang T. (2017). Dietary supplementation of enzymatically treated *Artemisia annua* could alleviate the intestinal inflammatory response in heat-stressed broilers. J. Therm. Biol..

[50] Park T.G., Kim Y.R., Park S.Y., Choi K., Kim K.J., Kim J.Y. (2023). Cinnamon (*Cinnamomum cassia*) hot water extract improves inflammation and tight junctions in the intestine in vitro and in vivo. Food Sci. Biotechnol..

[51] Gang G., Gao R., Li R., Jin X., Xing Y., Yan S., Xu Y., Shi B. (2025). Study on the regulatory effect of water extract of *Artemisia annua* L. on antioxidant function of mutton sheep via the Keap1/Nrf2 signaling pathway. Antioxidants.

[52] Gul B., Anwar R., Saleem M., Ahmad M., Ullah M.I., Kamran S. (2023). Attenuation of CFA-induced arthritis through regulation of inflammatory cytokines and antioxidant mechanisms by *Solanum nigrum* L. leaves extracts. Inflammopharmacology.

[53] Wang Z., Wang Z., Huang W., Suo J., Chen X., Ding K., Sun Q., Zhang H. (2020). Antioxidant and anti-inflammatory activities of an anti-diabetic polysaccharide extracted from *Gynostemma pentaphyllum* herb. Int. J. Biol. Macromol..

