# Supplementary material for: Development of Multi-Bioactive Driven Composite Plant Extracts and Functional Study in Mice and Piglets
Source: Antioxidants (Basel). 2026 Apr 9;15(4):468. doi: 10.3390/antiox15040468 (PMC13114034; doi:10.3390/antiox15040468)
Supplement: Supplementary file 1 [file antioxidants-15-00468-s001.zip › Table S1.pdf]

Table S1. Plant sources and prices of the 23 single extracts

Plant sources	Prices (CNY/kg)
Acanthopanax senticosus radix et caulis	130
Achyranthes bidentata radix	54
Artemisia annua	24
Artemisia scoparia Thunb.	30
Atractylodes macrocephala Koidz. rhizoma	54
Bambusoideae folium	44
Cinnamomum cassia presl	22
Citrus limon (L.) Burm. f. fructus	100
Cyperus rotundus L. fructus	30
Epimedium brevicornu maxim. folium	34
Euphorbia helioscopia L.	22
Filipendula palmate (pall.) maxim.	64
Fraxinus rhynchophylla Hance cortex	60
Ginkgo billoba L. folium	60
Glycyrrhiza uralensis Fisch. radix et rhizome	54
Gynostemma pentaphyllum (Thunb.) markino	44
Houttuynia cordata Thunb.	44
Ligustrum lucidum Ait. fructus	32
Magnolia officinalis cortex	52
Punica granatum L. pericarpium	20
Psoralea corylifolia L. fructus	40
Rosmarinus officinalis L.	88
Spatholobus suberectus Dunn caulis	32
