# Supplementary material for: Development of Multi-Bioactive Driven Composite Plant Extracts and Functional Study in Mice and Piglets
Source: Antioxidants (Basel). 2026 Apr 9;15(4):468. doi: 10.3390/antiox15040468 (PMC13114034; doi:10.3390/antiox15040468)
Supplement: Supplementary file 1 [file antioxidants-15-00468-s001.zip › Table S2.pdf]

Table S2. Factors and levels in the orthogonal experimental design.

Levels	Factors				
	AA, g	CCP, g	MOC, g	PGP, g	SSC, g
1	4	1	1	1	7
2	6	3	3	3	9
3	8	6	5	5	11
4	10	9	7	7	13

AA, *Artemisia annua*; CCP, *Cinnamomum cassia presl*; MOC, *Magnolia officinalis cortex*; PGP, *Punica granatum L. pericarpium*; SSC, *Spatholobi suberectus Dunn caulis*.
