# Supplementary material for: Development of Multi-Bioactive Driven Composite Plant Extracts and Functional Study in Mice and Piglets
Source: Antioxidants (Basel). 2026 Apr 9;15(4):468. doi: 10.3390/antiox15040468 (PMC13114034; doi:10.3390/antiox15040468)
Supplement: Supplementary file 1 [file antioxidants-15-00468-s001.zip › Table S3.pdf]

Table S3. Plant combinations generated using the orthogonal design.

Combinations	Factors				
	AA	CCP	MOC	PGP	SSC
1	1	1	1	1	1
2	2	2	1	2	2
3	3	3	1	3	3
4	4	4	1	4	4
5	2	3	2	4	1
6	1	4	2	3	2
7	4	1	2	2	3
8	3	2	2	1	4
9	3	4	3	2	1
10	4	3	3	1	2
11	1	2	3	4	3
12	2	1	3	3	4
13	4	2	4	3	1
14	3	1	4	4	2
15	2	4	4	1	3
16	1	3	4	2	4

AA, *Artemisia annua*; CCP, *Cinnamomum cassia presl*; MOC, *Magnolia officinalis cortex*; PGP, *Punica granatum L. pericarpium*; SSC, *Spatholobi suberectus Dunn caulis*.

Numbers 1-4 in the columns of AA, CCP, PGP, MOC and SSC represent the same levels as shown in Table S2.
