# Supplementary material for: Development of Multi-Bioactive Driven Composite Plant Extracts and Functional Study in Mice and Piglets
Source: Antioxidants (Basel). 2026 Apr 9;15(4):468. doi: 10.3390/antiox15040468 (PMC13114034; doi:10.3390/antiox15040468)
Supplement: Supplementary file 1 [file antioxidants-15-00468-s001.zip › Table S4.pdf]

Table S4. Ingredient composition and nutrient levels of the basal diet (as-fed basis)

Items	Content%
Ingredients	
Corn	59.53
Soybean meal	28.51
Wheat bran	3.0
Fish meal	1.0
Sugar	4.0
$CaHPO_4$	1.2
Limestone	1.0
NaCl	0.3
L-lysine	0.13
DL-methionine	0.03
Choline chloride	0.1
Premix[1]	1.2
Total	100.0
Nutrient levels[2]	
Crude protein	18.7%
Digest energy (MJ/kg)	14.55
Lysine	1.25
Threonine	0.75
Methionine+cystine	0.75

[1] The premix provided the following per kilogram of the diet: VA 5000 IU,VD_3 800 IU,VE 8 IU,VB_2 2.50 mg,VB_{12} 0.003 mg, biotin 0.15 mg, nicotinic acid 25 mg, folacin 0.5 mg, VK_3 0.5 mg, pantothenic acid 5 mg, thiamin 1.5 mg, VB_6 2 mg, Cu ($CuSO_4{\cdot}5H_2O$) 50 mg, Fe ($FeSO_4{\cdot}7H_2O$) 100 mg, Zn ($ZnSO_4{\cdot}7H_2O$) 100 mg, Mn ($MnSO_4{\cdot}H_2O$), 40 mg, Se ($NaSe_2O_3$), 0.3 mg, I (KI) 0.5 mg.

[2] Nutrient levels were calculated values.
