# Supplementary material for: Development of Multi-Bioactive Driven Composite Plant Extracts and Functional Study in Mice and Piglets
Source: Antioxidants (Basel). 2026 Apr 9;15(4):468. doi: 10.3390/antiox15040468 (PMC13114034; doi:10.3390/antiox15040468)
Supplement: Supplementary file 1 [file antioxidants-15-00468-s001.zip › Table S5.pdf]

Table S5. Results of range analysis of the bioactivities of extract combinations

Factors	Antibacterial rate%					Anti-inflammatory rate%					Antioxidant IC_{50} (mg/mL)				
	k_1	k_2	k_3	k_4	R	k1	k2	k3	k4	R	k1	k2	k3	k4	R
AA	36.6	55.3	33.9	48.6	21.5	78.6	72.4	71.0	72.0	7.52	0.54	0.62	0.59	0.79	0.26
CCP	40.1	35.4	46.7	52.1	16.7	76.3	76.0	71.1	70.6	5.74	0.45	0.54	0.93	0.62	0.48
MOC	28.5	28.8	56.8	60.3	31.7	69.8	68.5	78.4	77.4	9.90	0.68	0.44	0.84	0.59	0.4
PGP	58.4	45.4	45.3	25.2	33.2	82.5	73.8	69.5	68.3	14.2	1.15	0.69	0.38	0.32	0.84
SSC	45.3	43.4	29.3	56.4	27.2	65.2	72.7	75.3	80.9	15.8	0.53	0.84	0.65	0.52	0.32

K_1-k_4, the sum of the evaluation index values corresponding to under levels 1-4 of each factor, respectively; R, range value of each factor.
AA, *Artemisia annua*; CCP, *Cinnamomum cassia presl*; MOC, *Magnolia officinalis cortex*; PGP, *Punica granatum L. pericarpium*; SSC, *Spatholobi suberectus Dunn caulis*.
