# Supplementary material for: Development of Multi-Bioactive Driven Composite Plant Extracts and Functional Study in Mice and Piglets
Source: Antioxidants (Basel). 2026 Apr 9;15(4):468. doi: 10.3390/antiox15040468 (PMC13114034; doi:10.3390/antiox15040468)
Supplement: Supplementary file 1 [file antioxidants-15-00468-s001.zip › Table S6.pdf]

Table S6. Effects of extracts C1 and C2 on the relative abundance (%) of dominant colonic microbial community at the phylum level in mice.

Items	Treatments		
	CON	C1	C2
Firmicutes	58.02±2.568	56.22±1.340	53.92±1.240
Bacteroidetes	33.52±3.373	31.80±1.944	32.99±2.891
Proteobacteria	6.964±0.852 ^{A}	10.20±0.930 ^{B}	11.68±1.043 ^{B}

C1 and C2, extracts prepared from the different raw plant material ratio of *Artemisia annua* (AA), *Cinnamomum cassia presl* (CCP), *Magnolia officinalis cortex* (MOC), *Punica granatum L. pericarpium* (PGP), and *Spatholobi suberectus Dunn caulis* (SSC) , respectively.

Values are mean ± SE (n = 6).

Different capital letter superscripts in the same row indicate very significant differences (P < 0.01), no letter or identical letters indicate no significant differences (P>0.05).
