# Supplementary material for: Development of Multi-Bioactive Driven Composite Plant Extracts and Functional Study in Mice and Piglets
Source: Antioxidants (Basel). 2026 Apr 9;15(4):468. doi: 10.3390/antiox15040468 (PMC13114034; doi:10.3390/antiox15040468)
Supplement: Supplementary file 1 [file antioxidants-15-00468-s001.zip › Table S7.pdf]

Table S7. Effects of extracts C1 and C2 on the relative abundance (%) of dominant colonic microbial communities at the genus level in mice.

Items	Treatments		
	CON	C1	C2
Lachnospiraceae_NK4A136_group	28.43±2.026	28.74±1.689	23.93±1.336
Desulfovibrio	3.402±0.484	4.619±0.936	5.426±1.001
Alistipes	3.849±0.337	4.649±1.021	3.911±0.411
Helicobacter	3.223±0.720 ^{b}	5.072±0.343 ^{a}	5.668±0.718 ^{a}
Bacteroides	3.380±0.554	4.314±0.755	3.420±0.195
Lactobacillus	2.498±0.238	2.672±0.722	3.295±0.520
Roseburia	2.918±0.290	2.391±0.312	2.472±0.275
Prevotellaceae_UCG-001	0.688±0.489	0.247±0.018	0.295±0.040
Ruminococcaceae_UCG-014	0.925±0.120	1.040±0.199	1.010±0.106
Candidatus_Saccharimonas	0.281±0.055	0.548±0.291	0.365±0.073

C1 and C2, extracts prepared from the different raw plant material ratio of *Artemisia annua* (AA), *Cinnamomum cassia presl* (CCP), *Magnolia officinalis cortex* (MOC), *Punica granatum L. pericarpium* (PGP), and *Spatholobi suberectus Dunn caulis* (SSC) , respectively.

Values are mean ± SE (n = 6).

Different lowercase letter superscripts in the same row indicate very significant differences ($P < 0.05$), no letter or identical letters indicate no significant differences ($P > 0.05$).
